# Clusters of Nucleotide Substitutions and Insertion/Deletion Mutations Are Associated with Repeat Sequences

**DOI:** 10.1371/journal.pbio.1000622

**Published:** 2011-06-14

**Authors:** Michael J. McDonald, Wei-Chi Wang, Hsien-Da Huang, Jun-Yi Leu

**Affiliations:** 1 Institute of Molecular Biology, Academia Sinica, Taipei, Taiwan; 2 Institute of Bioinformatics and Systems Biology, National Chiao Tung University, Hsinchu, Taiwan; 3 Department of Biological Science and Technology, National Chiao Tung University, Hsinchu, Taiwan; Trinity College Dublin, Ireland

## Abstract

The genome-sequencing gold rush has facilitated the use of comparative genomics to uncover patterns of genome evolution, although their causal mechanisms remain elusive. One such trend, ubiquitous to prokarya and eukarya, is the association of insertion/deletion mutations (indels) with increases in the nucleotide substitution rate extending over hundreds of base pairs. The prevailing hypothesis is that indels are themselves mutagenic agents. Here, we employ population genomics data from *Escherichia coli*, *Saccharomyces paradoxus*, and *Drosophila* to provide evidence suggesting that it is not the indels per se but the sequence in which indels occur that causes the accumulation of nucleotide substitutions. We found that about two-thirds of indels are closely associated with repeat sequences and that repeat sequence abundance could be used to identify regions of elevated sequence diversity, independently of indels. Moreover, the mutational signature of indel-proximal nucleotide substitutions matches that of error-prone DNA polymerases. We propose that repeat sequences promote an increased probability of replication fork arrest, causing the persistent recruitment of error-prone DNA polymerases to specific sequence regions over evolutionary time scales. Experimental measures of the mutation rates of engineered DNA sequences and analyses of experimentally obtained collections of spontaneous mutations provide molecular evidence supporting our hypothesis. This study uncovers a new role for repeat sequences in genome evolution and provides an explanation of how fine-scale sequence contextual effects influence mutation rates and thereby evolution.

## Abstract 2

The authors propose that short repeat sequences may play an important role in causing the pervasive clustering of mutations across diverse genomes from prokaryotes to humans.

## Author Summary

An intriguing observation made during the comparison of genomes is that insertion and deletion mutations (indels) cluster together with nucleotide substitutions. Two (not mutually exclusive) hypotheses have been proposed to explain this phenomenon. The first postulates that an indel mutation causes an increase in the likelihood of the surrounding sequence incurring nucleotide substitutions, while the second claims that the region of DNA in which such a cluster is located is more likely to sustain both indels and substitutions. Here, we present evidence suggesting that the region of DNA, and not the indel, is associated with the accumulation of clusters of mutations over evolutionary time scales. We find that repeat sequences are closely associated with a large proportion of indels and that the abundance of repeat sequences is linked with regions of increased nucleotide diversity. By analysing molecular data and measuring the mutation rates of genes engineered to contain repeats, we find that the mutation rate can be manipulated by the insertion of long repeat sequences. On the basis of these results, we propose a model in which repeat sequences are prone to cause stalling of the high-fidelity DNA polymerase, leading to the recruitment of error-prone repair polymerases which then replicate the surrounding sequence with a higher-than-average error rate.

## Introduction

A major challenge of evolutionary genetics is to determine the mechanisms underlying cryptic patterns of mutation rate variation and how they influence evolutionary outcomes [1]. One of the most striking of these trends is the association between indel mutations and nucleotide substitutions [2]–[7]. Inter-species genome comparisons have revealed this trend to be universal to all prokaryotic and eukaryotic genomes examined thus far [4]–[6]. The prevailing explanation for this association is that indels, as “universal mutators” [4], cause the accumulation of nucleotide substitutions in the hundreds of base pairs of sequence surrounding the indel [4],[6]. Although such studies have been unable to unequivocally determine if the clusters are due to a single multimutational event (multiple mutation hypothesis), the indel per se (the mutagenic indel hypothesis), or the region of sequence in which the indel is found (the regional differences hypothesis), the mutagenic indel hypothesis has been adopted by workers in the field [8]–[12].

The mechanism of indel mutagenicity proposed by Tian and co-workers is that indels, when heterozygous, cause paired chromosomes to form heteroduplex DNA during meiosis [4]. This is posited to cause error-prone DNA repair systems to target indel-containing regions, leading to an increased likelihood of nucleotide substitution in the sequence surrounding the indel. Over time, this increase in mutation rate is predicted to leave as its signature the clustering of nucleotide substitutions in the DNA surrounding indels, while corresponding non-indel-containing orthologous sequences should have a lower number of substitutions, in accordance with the background substitution rate. In addition, because the proposed mutagenic effect of the indel is postulated to be dependent on its heterozygosity, the accumulation of substitutions should cease as soon as the indel becomes homozygous in the population. These predictions contrast with the regional differences hypothesis; regional effects are predicted to cause both indel and non-indel haplotypes to accumulate substitutions whether the indel is heterozygous or not. The multiple mutations hypothesis differs from both the regional and indel hypotheses in that clusters of mutations are due to a one-off mutation event. Determining whether mutations have accumulated over time or are due to a single mutation event is difficult without the ability to examine indel divergence on a temporal scale.

Here we use a population genomics approach to tease apart the dynamics of indel divergence using the genomes of *Escherichia coli*, *Saccharomyces paradoxus* (*S. paradoxus*), *Drosophila*, and humans. We show that it is not the indel but rather the sequence region in which the indel occurs that is associated with the accumulation of nucleotide substitutions over evolutionary time scales. We propose a mechanism whereby a DNA sequence that is prone to cause replication fork stalling causes the recurrent recruitment of error-prone DNA polymerases to certain DNA sites, resulting in an increased likelihood of nucleotide substitutions in the surrounding DNA sequence.

## Results and Discussion

To initiate our investigation into the mechanisms underlying indel-associated mutation, we used a unique population genomics resource: 20 high-quality genomes of the Escherichia/Shigella complex ranging from 0.1% to 2.5% sequence divergence (Table S1A). Employing this range of evolutionary distances facilitates capture of the incipient stages of indel divergence, minimizing the obscuring effect of time unavoidable during analyses of more diverged species. DNA replication and repair in *E. coli* are well understood and, due to their central and conserved role in all living cells, have provided a useful model for eukaryotic systems [13].

**Figure 1:**
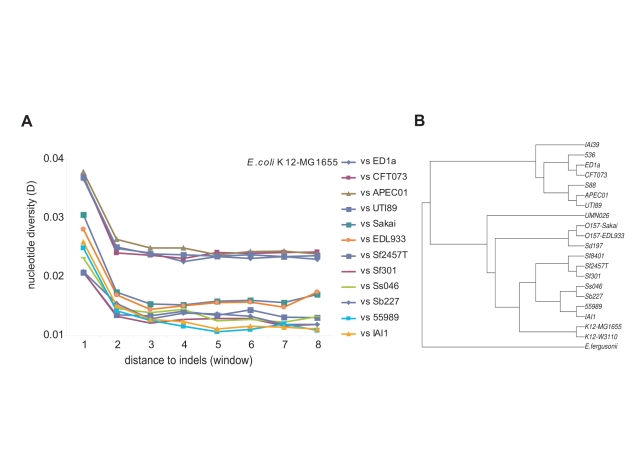
Nucleotide diversity increases with proximity to indels. (A) Twelve pairwise alignments between *E. coli* K12 MG1655 and other *E. coli* genomes are shown. Each number on the *x*-axis refers to a sequence window of defined size (Figure S1). The legend lists the comparisons in descending order of values of D for sequence window 1. A further six pairwise comparisons were omitted from this figure for clarity (see Table S4). (B) A phylogenetic tree constructed from 1,868 genes conserved in all 20 *E. coli* genomes used in this study as well as *E. fergusonii* (this phylogeny is adapted from Touchon et al., 2009 [34]).

Alignments were created between orthologous regions of pairs of *E. coli* genomes totalling 96.3 Mb, uncovering 5,390 indels. We then performed stringent tests to ensure that results were not due to artefacts of the alignment process (see Materials and Methods). Following Tian et al. [4], we generated estimates of overall nucleotide diversity, D, (D = 0.01 is equivalent to 1% divergence) and plotted the magnitude of D against sequence intervals of defined distance (designated as windows 1, 2, 3, etc.) from the nearest indel (Figure S1). Figure 1A shows an increase in nucleotide divergence in the sequence window closest to the indel (window 1) for all of the *E. coli* strain comparisons.

### Substitutions Accumulate Around Indels in Haploid (Non-Heterogenote) Bacteria

**Figure 2:**
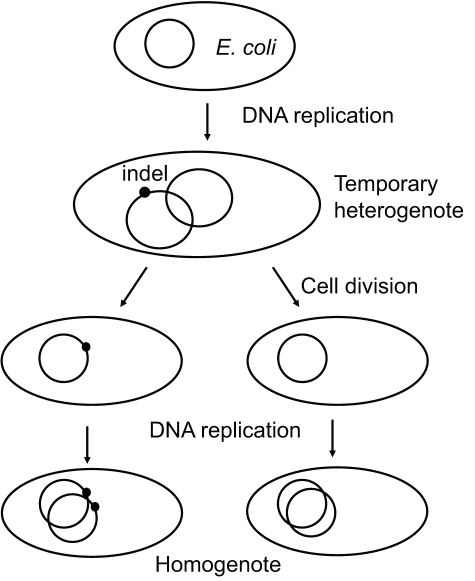
Indels in prokaryotes are only heterogenote for a short period of time between DNA replication and cell division. Cells have up to four copies of their genome during rapid growth. This raises the possibility that indels could be mutagenic during their attempted repair using the non-indel-containing chromosome copy. Following cell division, one of the daughter cells will possess an indel-containing chromosome, while the other daughter cell will not. The indel lineage will thereafter be homogenote for the indel. According to the previously proposed mutagenic-when-heterozygous hypothesis, the indel will not be mutagenic as it no longer exists as a heterogenote and nucleotide substitutions will not accumulate around them.

**Figure 3:**
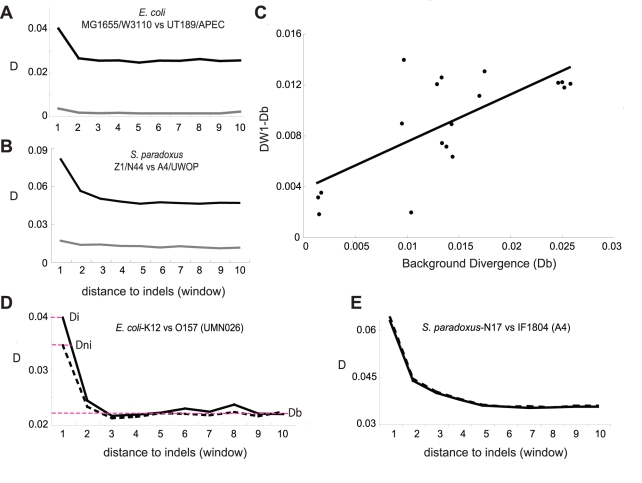
Indel-associated nucleotide substitutions accumulate over evolutionary time scales. Old indels (black) have accumulated a higher D than new indels (grey) in both *E. coli* (A) and *S. paradoxus* (B) (see Materials and Methods). (C) The indel-associated divergence (DW1-Db) is plotted against relatedness-associated divergence (Db) (as calculated for Table S4). DW1 is D of the window closest to the indel. Linear regression shows a significant correlation between background divergence (Db) and the value of DW1-Db (Pearson's correlation coefficient, *r* = 0.711, *p* = 0.00092). (D and E) Both indel and non-indel haplotypes have elevated D close to the indel containing site. Regions of indel haplotypes (solid lines) often have a higher value of D than regions of non-indel haplotypes (dashed lines) in sequence window 1, although this is never significant (two-sample Kolmogorov-Smirnov test, *p*>0.05, Table S2). (D) The analyses performed in *E. coli* and (E) the analyses performed in *S. paradoxus*. The strain used as the outgroup in each comparison is shown in parentheses. The total nucleotide diversity can be divided into fractions attributable to the indel + region effect (Di), the region (non-indel) effect (Dni), or the background level divergence of the two aligned orthologous fragments (Db) (red dashed lines in panel D).

The detection of indel-associated mutation in bacterial species poses a dilemma for the mutagenic-indel hypothesis. Prokaryotes are haploid; following the indel-causing event, the cell has only a brief heterogenote period during which, according to the mutagenic-when-heterozygous hypothesis, the indel is mutagenic. After a few cell divisions, the daughter cell will produce only indel-containing copies of the genome and will not have a non-indel version to recognize that the indel is present (Figure 2). The mutagenic-when-heterozygous theory then predicts (at least in prokaryotes) that nucleotide diversity does not accumulate over time. To test this prediction, we generated pre-defined, non-overlapping sets of old and new indels in *E. coli*. Old indels are those determined (using an appropriate outgroup) to have occurred before the divergence of the two strains under comparison; new indels are those that have occurred after their divergence (Materials and Methods, Figure S2). As shown in Figures 3A and S3, D values are significantly higher for old indels (black lines) than those for new indels (grey lines). This result demonstrates that, contrary to the mutagenic-when-heterozygous and multiple mutation hypotheses, mutations are accumulating at a higher rate in regions surrounding indels over time.

### Non-Indel Haplotypes Also Have Increased Amounts of Nucleotide Diversity

Background D (Db) is the average difference in the DNA sequences of two aligned orthologous regions. An increase in the number of differences between the nucleotide sequences of two aligned orthologous regions above this average indicates an increase in the rate of the accumulation of substitutions. The mutagenic indel hypothesis states that the indel per se is the cause of an increase in mutation rate and the accumulated nucleotide diversity in the surrounding sequence. A consequence of this is that, of two aligned fragments of DNA, the indel-containing fragment should have a highly elevated D close to the indel and its corresponding non-indel-containing orthologous fragment should have a D equivalent to the background. These predictions can be tested by choosing an orthologous sequence from a third *E. coli* genome as an outgroup to infer the ancestral state of the aligned sequence, thus allowing us to pinpoint in which of the two aligned genome fragments the indel event has occurred. This is dependent on the assumption of parsimony—if indels are a convergent character, the indel haplotype could be mistakenly assigned. D can be calculated for the sequence windows surrounding an indel-containing region (the indel haplotype) and the corresponding orthologous region without the indel (the non-indel haplotype) with which it is paired. In order to minimize the bias caused by differences in the selective constraints upon aligned sequences, we employed stringent filters to ensure that the sequences compared are strictly orthologous (see Materials and Methods). Figure 3D shows that the values of D for both the indel- and non-indel-containing haplotypes, Di and Dni, are elevated in window 1 as compared to the background nucleotide diversity Db. Although the values of Di in window 1 are often higher than Dni (an average 14% difference in D), this was not significant (two-sample Kolmogorov-Smirnov test, *p*>0.05, Table S2) for any of the strains compared. By contrast, when Di and Dni are compared to Db, in five out of six comparisons Di is significantly greater than Db (an average 57% difference in D), while Dni is significantly greater than Db in four cases (two-sample Kolmogorov-Smirnov test, *p* <0.05, Table S2; average 40% difference in D). Thus, for nearly as many instances as the indel haplotype, the non-indel haplotype has a D significantly higher than the background nucleotide divergence, confirming that the regional effect plays a role in the accumulation of nucleotide substitutions.

These results raise the possibility that the accumulation of mutations surrounding indels (Figure 3C) is mainly due to regional effects and not attributable to indels per se. However, this conflicts with the inferences of previous studies [2],[4],[6], that concluded that indels, not regions, are mutagenic. In order to find the cause of this disagreement, we took a closer look at the results of those studies as well as our own data. We noticed that the strains that are less diverged tended to have the largest difference between the indel and non-indel haplotypes (Table S2, Figure S4). Indels detected in the comparisons of two highly similar strains must have happened since their relatively recent divergence. The fact that the more diverged strains differed less between the indel and non-indel haplotypes suggests that the indel-associated effect diminishes over time. When we studied the results of [4] and [6], we found the same trend. For example, using data from [6], when bacterial divergence was plotted against difference between Di and Dni, it showed that the difference between Di and Dni decreases with increasing divergence (Figure S4). A further example is provided by Tian et al.'s [4] analysis of heterozygote alleles at one-third and two-thirds frequencies in yeast. The mutagenic-when-heterozygous mechanism predicts that indels occurring at a higher frequency in a population have been accumulating mutations for longer periods and should thus have a higher D value and a greater difference between Di and Dni. Conversely, the indels at two-thirds frequency have a smaller Di/Dni (1.40) than the indels at one-third frequency (2.23). The fact that indels that have been segregating for longer time have a smaller difference between the indel and non-indel haplotypes indicates that spending more time as a heterozygote actually diminishes the indel-associated effect, contrary to the prediction of the mutagenic-when-heterozygous hypothesis.

### The Proportion of D Attributable to the Indel Diminishes over Time

**Figure 4:**
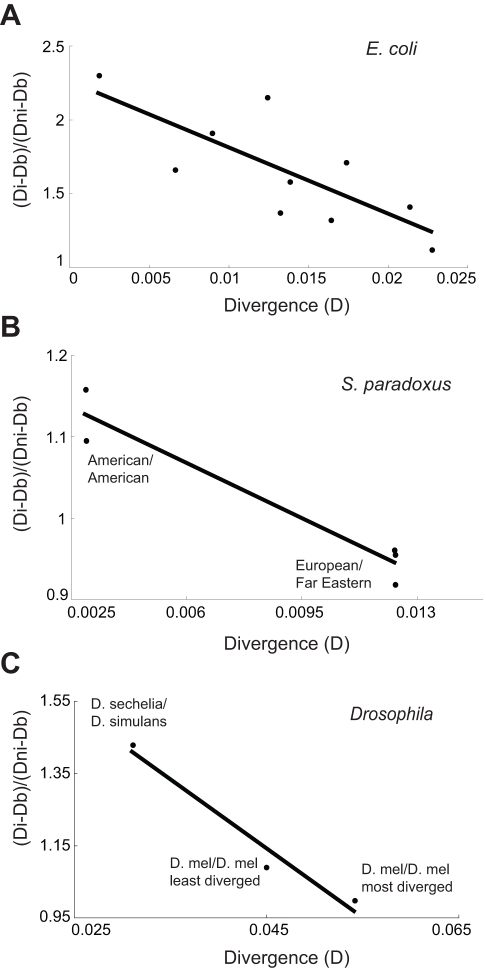
The proportion of D attributable to the indel decreases after the indel event. The indels found in comparisons between highly similar strains have a higher proportion of the nucleotide diversity attributable to the indel effect than sets of indels uncovered by pairwise comparisons of progressively more diverged strains for (A) *E. coli* (Pearson's correlation coefficient, *r* = −0.769, *p* = 0.00933), (B) *S. paradoxus* (Pearson's correlation coefficient, *r* = −0.963, *p* = 0.008), and (C) *Drosophila* (Pearson's correlation coefficient, *r* = −0.980, *p* = 0.128). Note that the pair *D. simulans*/*D. sechelia* is less diverged than the *D. melanogastor*/*D. melanogastor* comparisons because strains used for the latter were inbred with balancer chromosomes, allowing the accumulation of a large amount of mutations (see Materials and Methods).

The separation of D into Di and Dni allows us to calculate the proportion of D on the indel haplotype that can be attributed to the indel effect and to the regional effect, respectively (see Materials and Methods). Under the assumption that indel-causing events are uniformly distributed since the time of divergence, it follows that the level of divergence between two strains is correlated with the average age of the indels found during comparison. If an indel constantly influences the accumulation of nucleotide substitutions in the surrounding sequence while polymorphic, we expect to see an increase in the difference between Di and Dni over time. Conversely, if indels have a one-time-only effect on nucleotide diversity, we expect to find a decline in this difference over time. We compared Di and Dni for alignments identifying new and old indels (Materials and Methods, Table S3). Figure 4A shows that the difference between Di and Dni decreases with increasing divergence (Pearson's correlation coefficient, *r* = −0.769, *p* = 0.0093). This negative correlation is striking when compared to the positive correlation between time since divergence and nucleotide diversity when the indel and region effects are not separated (Figure 3C, Pearson's correlation coefficient *r* = +0.711, *p* = 0.00092). This result suggests that it is the region, but not the indel, that is constantly influencing the accumulation of substitutions over evolutionary time scales.

### Patterns of Indel-Associated Divergence Identified in Prokaryotes Hold True for Uni- and Multi-Cellular Eukaryotes

To test whether the aforementioned phenomenon is specific to prokaryotes, we carried out analogous indel analyses using the budding yeast *Saccharomyces paradoxus.* This organism is suitable for analysis because genome sequences are now available for a variety of its strains [14] and because *S. paradoxus*, like many multicellular eukaryotes, spends most of its life as a diploid [15]. The results of the analyses with *S. paradoxus* (Figures 3B, 3E and 4B, Table S3) were in agreement with those obtained using *E. coli* sequences. The *S. paradoxus* strains used here (Table S1B) cover a wider range of divergence than the *E. coli* strains [16]; this allowed us to view the diminishing proportion of the indel-dependent component of D on a longer time scale (Figure 4B, Pearson's correlation coefficient *r* = −0.963, *p* = 0.008). We then extended our analysis to *Drosophila* species (Figure 4C) (see Materials and Methods). Although few species diverged recently enough to be suitable for analysis, the results corroborate our prior findings that the proportion of D attributable to the indel decreases over time (Pearson's correlation coefficient *r* = −0.980, *p* = 0.128). It should be noted that the ratio of (Di − Db)/(Dni − Db) was calculated for several yeast and fly alignments with greater divergence than shown in Figure 4; in all cases, this ratio was approximately one (Table S3). All these results suggest that a difference between the indel and non-indel haplotype exists following the indel-causing event but that this difference decreases over time until stabilising with both haplotypes having the same amount of nucleotide diversity. Because our study is able to track indel divergence within a species, this analysis provides unequivocal evidence that nucleotide diversity associated with indels decreases over time.

### Indel-Associated Nucleotide Substitutions Bear the Signature of Error-Prone DNA Repair Enzymes

**Figure 5:**
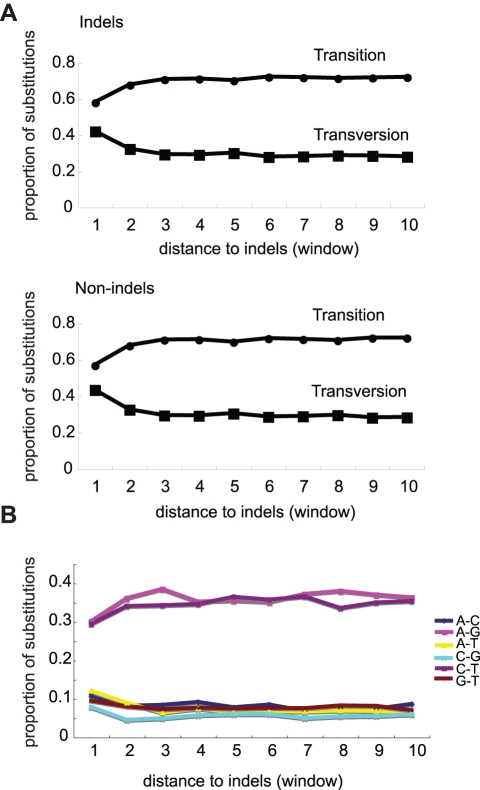
Transversion mutations comprise a larger proportion of all mutations close to indels. Transition mutations are usually the most common type of mutations, as indicated by their prevalence in the regions of sequence outside the influence of indel/region-associated mutagenicity. (A) Comparison of indel and non-indel haplotypes reveals that both exhibit the same increase in transversion substitutions with increasing proximity to the indel site. The difference between the number of transitions close to the indel (window 1) was found to be significantly lower than in sequence further from the indel (window 4) (two-sample Kolmogorov-Smirnov test, *p*<0.0001). (B) The proportions of each type of transversion (bottom lines) and transition (top lines) as a function of indel position. Transitions and transversions given in the legend represent substitutions in both directions (i.e., A–G includes both A–G and G–A transitions).

**Figure 6:**
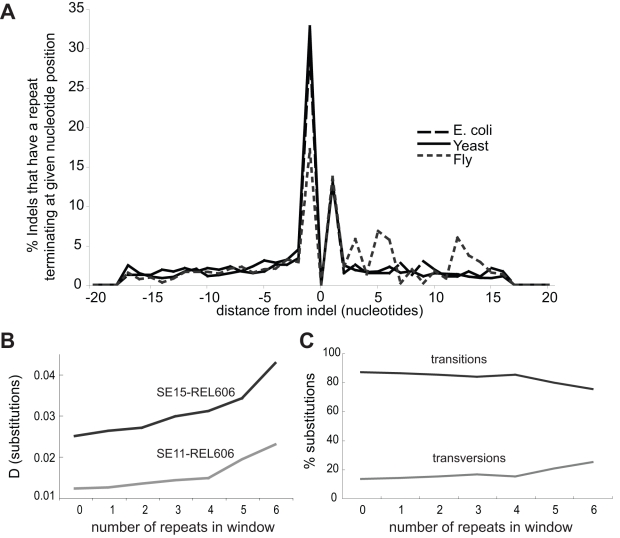
Repeat-rich sequence windows contain increased sequence diversity. The location of repeat sequences often coincides with indel position in *E. coli*, *S. paradoxus*, and *Drosophila*. Shown are the 20 nucleotides upstream (negative integers) and downstream (positive integers) of the indel (position zero). A repeat is scored once in the nucleotide position in which it terminates, for example, A repeat of four A's running from position −5 to −1 is recorded as a repeat at position −1 (A). Sequence windows from new *E. coli* alignments not containing indels were binned according to the number of 4 mer homonucleotide repeats they contained. D was found to increase with the increased number of repeats (B), as did the ratio of transversion to transition substitutions (C). The repeat density effect was stronger in a more diverged two-strain comparison (B), indicating that repeats are associated with the accumulation of substitutions over time.

Mutations arise from inaccurate processing of DNA damage or errors incurred during DNA replication. *E. coli* possesses five DNA polymerases of which two, Pol IV and Pol V, are error-prone. These polymerases are recruited to stalled replication forks [17],[18] and double-strand breaks [19] to restart DNA replication. Errors made by DNA Pol IV are biased towards frameshifts [20], and though genomes exhibit a bias towards transitions [16], DNA Pol V most often causes transversion mutations [21]–[23]. We analysed the ratio of transition to transversion changes for all aligned *E. coli* genomes and found that transversions are enriched close to indel and non-indel haplotypes (two-sample Kolmogorov-Smirnov test, *p* <0.0001) (Figure 5); this is also true for *S. paradoxus* and other eukaryotes [4]. The accumulation of mutations at a specific site at a higher rate is uncharacteristic of mutations caused by a mutagenic chemical or another random event and is most likely due to the persistent recruitment of error-prone polymerases to that site over evolutionary time. Impediments imposed by polynucleotide repeats or other repeat sequences are suggested to be common causes of DNA replication fork arrest [24]. We performed a computational analysis on the 20 bp immediately flanking our collection of *E. coli*, *S. paradoxus*, and *Drosophila* indels to determine the distribution of repeats around indels. We defined an indel as contiguous with a repeat if it occurred inside or immediately next to a repeat, and as repeat-proximal if some part of a repeat was positioned within 5 bp on either side of the indel. For *E. coli*, 43% of indels were contiguous with a homopolymer, while 20% were proximal. The corresponding numbers were 45% and 25% for yeast and 31% and 34% for flies, respectively (Figure 6A).

### The Repeat-Sequence-Induced Recurrent Repair (3R) Hypothesis

The association between repeat sequences and indels is well understood: repeat sequences are prone to sustain strand slippage mutations [25],[26], which tend to cause indels [19],[27]. We propose a mechanism distinct from strand slippage for the regional increase in nucleotide substitutions, whereby repeat sequences and other polymerase-stalling motifs persistently cause the recruitment of error-prone DNA polymerases. Each time DNA replication is restarted by an error-prone polymerase, DNA surrounding the region will be synthesized with a higher rate of error [17],[18],[28], leading to an increased likelihood of nucleotide substitution. The stalled fork also suffers a high rate of double-strand breaks, another route to error-prone repair [19],[27],[29]. The 3R hypothesis predicts that regions of a genome with increased sequence diversity should be able to be identified by repeat sequence abundance. We tested this prediction by using the recently sequenced genomes of three *E. coli* strains that we had previously not analysed. We searched for repeat-rich regions by first generating pairwise alignments as for our indel analysis, dividing these into non-overlapping 100-bp windows, and then binning each window according to its number of 4-nucleotide homopolymer repeats (see Materials and Methods). We found that, even when indel-containing windows were excluded, windows with a higher number of repeat sequences had more nucleotide substitutions than those without (83% increase for SE11/REL606 and 71% increase for SE15/REL606 in windows with six repeats). As for indel-based analyses, the more diverged two-strain comparison had a higher value of D, supporting that repeats cause the accumulation of substitutions over time (Figure 6B). We also found that the number of transversions relative to transitions was increased in repeat-rich regions (88% increase in windows with six repeats) (Wilcoxon Sum Rank, *p*<0.05, Figure 6C, Table S5).

### Mutagenic Indels?

The “bump” in nucleotide substitutions associated with the indel (the difference between Di and Dni) that we and others [4],[6] often observe requires an explanation. The declining ratio of Di/Dni shows that this bump is smoothened over evolutionary time (Figure 4). One explanation for this is that indel mutagenicity is transient because the indel-containing allele is only mutagenic as a heterozygote and its mutagenic effect will vanish when it becomes homozygous. The period for which bacteria exist as heterogenotes for an indel is orders of magnitude less than that for diploid eukaryotes. However, a consistent decrease in Di/Dni is found across taxonomic kingdoms, an observation at odds with the proposal that heterzygosity/heterogenosity causes the indel “bump.” An alternative explanation is that the indel-associated bump in D may be due to the indel-causing event resulting in multiple nucleotide changes. This possibility is not implausible considering the spectrum of mutations in baker's yeast. Lang and Murray [30] found that in 63% of instances where two mutations occurred at the same time one was an indel and the other a nucleotide substitution; yet indels constituted only 6.67% of all mutations observed in that study. Whichever explanation is correct, it is evident that the indel effect is transient and that it is the surrounding sequence that is associated with the accruement of substitutions over evolutionary time scales.

### Indels, Substitutions, and Repeat Sequences Collected from a Haploid, Non-Indel-Containing *ura3* Marker Cluster Together

All the inferences made about indels, nucleotide substitutions, and repeat sequences have so far been drawn only from the comparisons of genomes. In order to test predictions made by the 3R and mutagenic indel hypotheses, we utilized the comprehensive collection of spontaneous *ura3* mutants gathered by Lang and Murray [30]. This collection comprises 207 *ura3* mutant alleles, each of which resulted from a single mutational event in a haploid (and non-indel-containing) gene. The mutagenic indel hypothesis predicts that the clustering of mutations is caused by indels; thus, this set of independently occurring mutants should not cluster. Conversely, the 3R hypothesis states that repeat sequences cause an increase in the likelihood of the surrounding sequence sustaining both indels and nucleotide substitutions; thus, according to this hypothesis, indels and substitution mutations collected from independent mutants should cluster around repeats. Using a model based on a hyper-geometric distribution (Materials and Methods), we first found that indels and substitutions cluster together (*p* = 0.019), even though most substitutions occurred without a co-occurring indel (97%). Next, we tested for the association of indel/nucleotide substitution mutations with any of the 264 four-nucleotide combinations of A, T, C, and G (e.g., ATCG, ATCA, ATCT, etc.). It is expected by chance that 2 or 3 four-nucleotide combinations should be found to be significant; however, significant associations were found only with the repeat sequences TGTG (*p* = 0.00027), AAAA (*p* = 0.0093), and GTGT (*p* = 0.0098). These results confirm that indels, substitutions, and repeat sequences are associated independently of any initiating mutator indel.

### Experimental Determination of Repeat-Induced Increase in Mutation Rate

**Figure 7:**
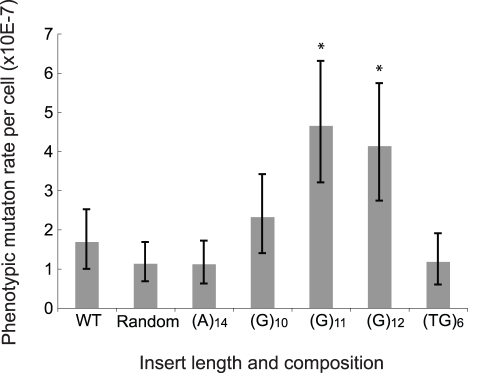
Insertion of repeat sequences upstream of *URA3* increases the mutation rate to Ura−. 12-nucleotide insertions were engineered four base pairs upstream of *URA3*. Mutation rates of different insertions were determined by fluctuation test using at least 10 cultures. Data represent the mean of three repeats. The strain denoted as wt has no insertion and that designated as random has a non-repeat 12-nucleotide insertion (see Materials and Methods for the sequence). Error bars represent 95% confidence intervals. Significance was calculated using *t* tests, and asterisks indicate *p*<0.001.

We directly tested whether insertions of repeat sequences could increase the mutation rate of nearby regions in yeast. We engineered a copy of the *URA3* gene to contain either a poly(A) repeat, a poly(G) repeat, a poly (TG) repeat, or a random 12-mer sequence in the promoter, verified that these constructs did not abolish *URA3* function, and then performed fluctuation tests using the maximum likelihood method to determine the mutation rate to *URA3* inactivation. We observed that (G)11 and (G)12 conferred a significant increase in the phenotypic mutation rate compared to the wild type (paired *t* test, *p*<0.001, Figure 7). Insertion of a shorter poly(G) sequence also conferred an increased rate, but the changes were less significant. On the other hand, the insertion of a random 12-mer sequence, poly (A), and poly (TG) showed no effect on the mutation rate. The fact that poly(G) causes an increase in the mutation rate is interesting considering that tetranucleotides composed of G or C bases are absent in the *URA3* gene and are 5–10-fold less common across *E. coli*, *S. cerevisiae*, and *Drosophila* genomes than A or T tetranucleotides (unpublished data).

### Indel Divergence in Human Transcribed Sequences

**Figure 8:**
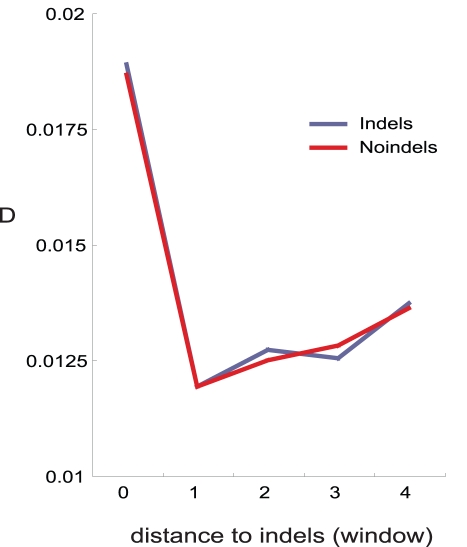
Human transcribed sequences accrue nucleotide substitutions in the sequence surrounding indels and their corresponding non-indel orthologous regions. Recent duplications (<5% divergence) were aligned and non-duplicated orthologous regions from the Chimp genome were used as outgroups to allow identification of indel and non-indel haplotypes.

In order to determine if clusters of indels and substitutions influenced coding sequences in humans, we used alignments of recent segmental duplications (<5% diverged) [31] to detect indels in the human genome, restricting our analysis to those sequences that had been confirmed as expressed (see Materials and Methods). We found that indels and nucleotide substitutions occurring in human transcribed sequences follow the same patterns observed in other species, confirming that indel/region/repeat-associated mutation impacts genes expressed in humans (Figure 8).

### Conclusion

Here we have provided evidence suggesting that regional effects have a strong influence on the accumulation of nucleotide substitutions over evolutionary time scales. Although an indel effect is also observed, we have shown the proportion of D attributable to an indel effect diminishes over time. In addition, it is not possible to formally exclude whether this effect is due to a mutagenic indel effect or a single multiple mutation causing event.

Although we found that many indels are associated with repeat sequences, many are not. This finding may be explained by the existence of other non-repeat polymerase stalling sequence motifs; another possible explanation is that repeat sequences were destroyed by mutation, while the indel remained.

So what is the impact of the indel/region effect on phenotypic evolution? Most indels in *E. coli* are within 100 bp of the nearest gene (Figure S5). In *S. cerevisiae*, 25% of promoters contain repeat sequences [32] and 600 seven-nucleotide homopolymer runs have been identified in essential genes [33], putting cis-regulatory regions and coding sequences well within the range of the effect of indel/repeat-associated mutation.

## Materials and Methods

### Sequences and Alignments

The genomes and accession numbers used for *E. coli*/*Shigella* and *S. paradoxus* analyses are shown in Table S1. Genome sequences for alignments between Drosophila species were downloaded from the UCSC database (http://www.biostat.wisc.edu/~cdewey/fly_CAF1/), while those for melanogastor/melanogastor alignments were downloaded from http://www.dpgp.org. The alignments of recent human segmental duplications were provided by [31]. For pairwise comparisons, genome sequences were aligned using BLAST with default parameters and divided into orthologous regions of at least 3 kb in length and >80% nucleotide sequence identity. Any region that could be aligned to multiple locations was not considered for analysis, ensuring that only orthologous sequences were used. A program was written in Perl script to find indel mutations within orthologous regions; those regions not containing indels were discarded. For three and four genome alignments, orthologous regions that were not common to all strains were discarded and those regions remaining were realigned using ClustalW.

### Indel/Non-Indel Analysis

In order to determine in which of two aligned fragments an indel has occurred, an appropriate outgroup was selected using the phylogenetic tree [34] and confirmed by our own approximations of relatedness (Table S4). In addition to establishing in which of the fragments the indel had occurred, the number of nucleotide substitutions occurring in the indel containing haplotype (Di) and non-indel containing haplotype (Dni) was determined by comparison with an outgroup sequence. For instance, when three genomes were aligned to determine indel and non-indel haplotypes, the number of mutations on the non-indel haplotype was counted by comparison of the non-indel fragment with the outgroup, and the number of substitutions on the indel haplotype was calculated by comparing the indel haplotype and the outgroup. Statistical comparisons between indel- and non-indel-containing haplotypes were carried out using the non-parametric Kolmolgorov-Smirnov paired test. See the statistical analysis plan below for more details.

### Repeat Sequences

An indel was designated as contiguous with a repeat for cases where the indel occurred inside the repeat (A-AAA, AA-AA, or AAA-A), or immediately next to it (−AAAA or AAAA−) where − denotes the position of the indel. It was defined as near a repeat if any part of a repeat was within five nucleotides on either side of the indel (AAAANNN−, AAAAN−, etc.). For the search for regions of high D on the basis of repeat sequence density, we used three *E. coli* strains not previously used in this study (*E. coli* SE11, *E. coli* SE15, and *E. coli* B Str. REL606). We searched for repeat-rich regions by first generating pairwise alignments (as described for the indel analysis above), followed by generating non-overlapping 100-bp windows and binning of windows according to the number of homopolymer repeats of at least 4 nt in length. Repeat sequences interrupted by a substitution mutation so that the homopolymer was less than four continuous nucleotides in length were not included. We then calculated total D for each window as well as the D for these classes of mutation: substitution, indel, transition, and transversion. To test for statistically significant differences between different classes or 100-bp windows, we used the Wilcoxon Sum Rank test.

### Analysis of Aligned, Indel-Flanking Sequences

In order to extract indel-flanking sequences for analysis, the positions of indels were recorded in each orthologous region. Next, the sequences (1 kb) both up- and downstream were extracted and examined for additional indels. If one of the flanking sequences was found to contain additional indels, that flanking region was discarded. The sequence surrounding the indel was named and ordered into windows (Figure S1). For every analysis in this study, the nucleotide divergence (D) was calculated for each window using the Jukes-Cantor method [35].

### Old and New Indels

Pairs of recently diverged strains were chosen based on a phylogenetic tree (Figure 1B). Each of these designations as highly related was supported by our own estimations of divergence provided by pairwise alignments (Table S4). Two pairs of recently diverged strains were aligned by performing a new alignment of all four orthologous fragments in ClustalW, giving a total of four aligned genomes. New indels were those that occurred within pairs of recently diverged strains; for indels to be detectable, they must have occurred since the recent divergence of these two strains (see Figure S2). D for new indels was calculated using the alignment of two similar strains, of which one had been found to contain the indel. Old indels were those sites which concurred within recently diverged pairs but were different between the two pairs (see Figure S2). Such indels must have happened before the divergence of the highly similar strains yet after the divergence of the two sets of strains. For calculating D, one from each of the sets of similar genomes was selected, so that two highly diverged genomes were compared and from this comparison D is calculated for old indels. If there are double mutations (sites where the two similar genomes are different from each other and the other diverged pair), these are scored as one substitution because the difference between the two similar strains must have happened since the divergence of the two diverged sets of strains and have already been scored in the new-indel analysis. The background divergence (Db) used for the regression shown in Figure 3C was calculated as the average D from windows 3 to 10 for each *E. coli* pairwise alignment (window 1 comprises the 50 bp closest to the indel; windows 3 to 10 were assessed as consistently outside the range of influence of the indel) (see Figure 1A). The indel-associated divergence was calculated by subtracting the values obtained for Db from the value of D at window 1.

### Statistical Analysis Plan for Pairwise Comparisons of Indel and Repeat Data

For pairwise comparisons between indel and non-indel haplotypes, previous studies have used paired *t* tests, however we found that our data was not normally distributed (Shapiro-Wilk test for normality, *p*<0.05). We used the two-sample Kolmogorov-Smirnov test to test for the appropriateness of the non-parametric Wilcoxon Sum Rank test for our samples. If the samples were found to be different by the Kolmogorov-Smirnov test, the Kolmogorov-Smirnov test was named and *p* value given (as was the case for the indel/non-indel analysis). If the two-sample Kolmogorov-Smirnov test found the samples under comparison to be of the same shaped distribution, we carried out and presented the Wilcoxon Sum Rank test and *p* values (this was the case for the repeat/window analysis).

### Correlation of D and Age of Indel

A comparison of the amount of nucleotide substitutions attributable to the indel and regional effects for indels of different ages would provide for a test of the hypothesis that indel-associated mutations accumulate over time. In principle, this could be achieved by using the sets of old and new indels used for the analysis presented in Figure 3A and 3B; however, the generation of the set of old indels required a four-genome alignment; a fifth genome needs to be added to determine the indel and non-indel haplotypes. Because of our strict criteria for defining orthologous regions, the partitioning of the old and new indel sets into indel and non-indel haplotypes leaves prohibitively few orthologous regions for analysis. An alternative is to consider pairwise sets of alignments. The background nucleotide diversity for each pairwise comparison (Figure 1) provides a measure of relatedness; the greater the average value of background D, the more diverged the two strains. In order to gauge the range and degree of difference across these pairwise comparisons, the sets of background diversity values (provided by the D values for windows 3 to 10, which were chosen because they are outside the range of indel/region-associated influence) were compared. We found that most strains had distinct levels of sequence divergence from each other (Tukey's HSD, *p*<0.05, Table S4), with an approximately 20-fold difference in D values between the most and least diverged strains (see Table S4 for details). In order to cover a range of pairwise comparisons of increasing divergence, we chose four strains and systematically compared them to strains from clades of increasing divergence. The least divergent outgroup was always chosen. Each value of D can be partitioned into composite fractions (Figure 3D and 3E). Di is attributable to the effect of the indel and the region together, whereas Dni is attributed to the region alone. (Di − Db)/(Dni − Db) provides a measure of the total proportion of Di that is influenced by the indel. If (Di − Db)/(Dni − Db)  = 1, none of the increase in nucleotide diversity can be attributed to the indel. As the value increasingly exceeds one, more of the nucleotide substitutions surrounding indels can be attributed to the indel effect. The indels detected in pairwise comparisons of more diverged strains cannot be strictly called “old” indels; these pairwise alignments will also include indels that have occurred relatively recently. However, increasingly divergent strains will be composed of a greater proportion of relatively old indels. This method of comparing indels between less diverged and more diverged strains will therefore underestimate the negative association between indels and the accumulation of nucleotide substitutions.

### *D. melanogastor/D. melanogastor* Indel Analysis

In order to explore indel divergence in a metazoan genus, we aligned sequenced genomes of the genus Drosophila. However, all pairwise comparisons (except the alignment of *D. sechelia* and *D. simulans*) were diverged so much that the difference between Di and Dni was undetectable ((Di − Db)/(Dni − Db) = 1). To possibly obtain alignments of less diverged genomes, we used alignments of 37 genomes available from the *D. melanogastor* 50 genome project (http://www.biostat.wisc.edu/~cdewey/fly_CAF1/). However, the alignment of any two of these genomes could not give enough indels suitable for analysis; most indels detected within *D. melanogastor* tended to cluster, leading to the rejection from our analysis of many indel-containing regions. To overcome this, suitable indels found from the alignment of all 35 strains from the Raleigh collection [36] to two of the Malawi strains (MW63 and MW27) [37] were used; indels found in more than one alignment were discarded, and from this set the 100 most and 100 least diverged indel-containing alignments were taken (background divergence was taken as Db and calculated based on the average D of windows 3 to 10).

### Modelling the Distribution of Indels, Nucleotide Substitutions, and Repeat Sequences Using a Hypergeometric Distribution

Each nucleotide site of *URA3* was classified as being mutable or not, based on the 5 bp of sequence on each side of that nucleotide, creating a stringent null model for the expected distribution of nucleotide substitutions and indel mutations. The probability of obtaining the observed distribution under the null model was calculated using the hypergeometric distribution: 
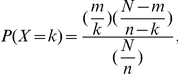



where for the test for association between indels and substitutions, *m* is the total number of windows which are defined as mutable, *k* is the number of times an indel is in a region defined as mutable, *N* is the number of sliding windows, *n* is the total number of indel mutations, and for the test for association between repeat sequences and indels and substitutions, *k* is the number of times a tetranucleotide sequence *x* is contiguous with a nucleotide site defined as mutable and *n* is the total number of times a tetranucleotide sequence *x* appears in *URA3*.

### Mutation Rate Analysis

A single (TG)_6_, (G)_12_, or (A)_12_ tract (or a random 12-mer (AAGTGTCAAATA) as a control) was inserted between positions −4 and −5 of *URA3*. Because these sequences are inherently unstable, multiple lengths of a homonucleotide tract were recovered during the cloning process, all of which left *URA3* functional—providing evidence that alteration in the length of this sequence could not confer the Ura-, 5-FOA-resistant phenotype. Fluctuation tests were carried out in order to determine the mutation rate of altered *URA3* genes. These were carried out by first setting up overnight cultures of each strain to be assayed in CSM-Ura media to ensure maintenance of the functional *URA3* gene. The following day each strain's culture was diluted so that low numbers of cells (∼1,000) were inoculated into at least 10 independent 100 µl YPD cultures per strain in 96 well plates. Cultures were incubated at 30°C for 2 d without shaking and then spot plated onto dry 5-FOA plates. Aliquots (5 µl) of each culture were pooled, diluted, and subsequently plated onto three YPD plates to determine the total cell count. Each experiment was repeated three times. Mutation rates were calculated using the equation µ = m/Nt, where m is the mutant frequency and Nt is the total number of cells in the culture. m was determined by counting the number of 5-FOA resistant colonies for each of the 3 sets of 10 independent cultures; then calculations were carried out using FALCOR software [38] (http://www.keshavsingh.org/protocols/FALCOR.html#interface), which employs a maximum likelihood method developed by Sarkar, Ma, and Sandri [39]. The resultant value for m (mean mutant frequency) is divided by the total number of cells in the culture Nt. Nt provides a measure of the total cell divisions that have occurred in the culture; therefore, our final unit is number of Ura− mutants per cell division. Error bars are 95% confidence intervals as calculated by FALCOR using a formula devised by [40]. *t* tests were used to compare all strains to the wild-type strain, using formula 5 on the FALCOR website.

### Human-Human Indels

In order to identify indels occurring within the human lineage that may have influenced phenotypic evolution, we used a collection of recent segmental duplications (<5% diverged) [31] and identified them as expressed by comparing with the human mRNA sequence collection (refseq, NCBI). We used the Chimpanzee genome as an outgroup to identify indel and non-indel haplotypes (http://hgdownload.cse.ucsc.edu/downloads.html#chimp). All human segmental duplications were present as a single copy in the chimpanzee genome. The non-indel haplotype corresponds to the human copy that is the same as the chimp single copy at the indel site, while the indel-containing copy is the one that differs from the chimp version at the indel site.

### Comparison of Indel Position and Potential Sequence Elements of Interest

We searched for an association between indel sites and various sequence elements that could have been associated with an increased nucleotide substitution rate. We generated a list of indels found in the *E. coli* K12 MG1655 genome, the best studied of all *E. coli* strains for which such sequence elements are well characterized. For each indel, the sequence region flanking 1 kb of the indel was designated as an indel-containing portion of the genome. The frequency with which sequence elements of interest were found in indel-containing portions of the genome compared to the rest of the genome was scored. The sequence elements that were searched were transposable elements and insertion sequences, tRNA genes, recombination sites (as indicated by the chi site), DNA sites prone to breakage (sites identified by the program Twist Flex), and repeat sequences.

## Supporting Information

Figure S1 Sequence intervals for indel-flanking regions. In order to extract indel-flanking sequences for analysis, the positions of indels were recorded in each orthologous region. Next, the sequences (1 kb each) on each side of the indel were extracted and examined for additional indels. If one of the flanking sequences was found to contain additional indels, that flanking region was discarded. Blocks of sequence regions surrounding a specific indel are named and ordered as windows 1 to 10 (W_1_–W_10_): W1 comprises the 50 nucleotides closest to the indel, W_2_–W_9_ are each composed of 100 nucleotides, and W10 consists of the outermost 150 nucleotides. In each window, the nucleotide divergence is computed by the Jukes-Cantor method. The method of calculating D and the window sizes are as used by Tian et al. [4]. See Figure S6 for analyses using alternative window sizes.
(0.40 MB EPS)

Figure S2 The generation of distinct sets of old and new indels. Orthologous regions of highly related strains A1 and A2 were aligned with another set of highly related strains B1 and B2. Old indels are defined as indels that happened before divergence of the closely related species. For example, if B1 and B2 both have the same indel but A1 and A2 both do not, this would be considered an old indel, as it must have happened before the divergence of the highly similar strains B1 and B2. Conversely, if an indel is present only in A1 but not A2, B1, or B2, this indel is new because it must have happened after the divergence of A1 and A2.
(0.41 MB EPS)

Figure S3 Indel-associated nucleotide substitutions accumulate over evolutionary time scales. Old indels (black) have accumulated a higher D than new indels (grey) (A–D). MG1655/W3110 versus CFT073/ED1a refers to a four-genome alignment of the recently diverged K12 MG1655 and K12 W3110 to the recently diverged strains CFT073 and ED1a (see Materials and Methods). All statistical tests in this study are two-tailed.
(0.46 MB EPS)

Figure S4 The difference between Di and Dni decreases with divergence in a wide range of bacteria. Figure based on an analysis using original data from [6], bacterial species and data given in Table S6.
(0.43 MB DOC)

Figure S5 The distribution of the distances of indels relative to protein coding genes in *E. coli*. The positions of all indels found in this study were determined in annotated genome sequence to calculate their location relative to genes.
(0.44 MB EPS)

Figure S6 Indel-associated mutation using alternative window sizes. Two strain comparisons, old/new indel and indel/non-indel analyses, were repeated using either all 50-nucleotide windows or 100-nucleotide windows in *S. paradoxus*. A representative sample is shown here.
(0.47 MB EPS)

Table S1 Bacterial and yeast strains used in this study. (A) Genomes of *Escherichia coli* strains used in this study. (B) Yeast strains used in this study. The accession number is given according to the internal collection at the University of Nottingham. Strains are grouped into geographic locations from which they were isolated (for a detailed phylogeny of *S. paradoxus* strains used in this study, see Liti et al. 2009 [14]).
(0.05 MB DOC)

Table S2 The accumulated amount of nucleotide substitutions in indel haplotypes is rarely significantly higher than the amount in non-indel haplotypes in the sequence window closest to the indel (window 1). The values of D for the indel- and non-indel-containing haplotypes for window 1 were compared using the two-sample Kolmogorov-Smirnov test (n is the number of indel/non-indel pairs used in the analysis). Indel and non-indel haplotypes have elevated nucleotide divergence in window 1 as compared to the background level of divergence (Db). The values of D for window 3 were chosen to represent Db; this level was compared with the level in window 1 to determine if there was a significant increase in nucleotide substitutions for both the indel and non-indel haplotypes by performing two-sample Kolmogorov-Smirnov tests. Significant values for *p* (*p* < 0.05) are indicated in bold. n is the number of indel/non-indel pairs used in the analysis.
(0.04 MB DOC)

Table S3Divergence values for indel and non-indel haplotypes. (1) D is the average divergence between the entire genomes of strains being compared. Di and Dni denote the divergence in sequence window 1 of the indel-containing and non-indel-containing haplotype, respectively. Db is the background level of diversity as measured by sequence windows 3 to 10. (2) Outgroups were used to determine in which of the aligned genomes the indel had occurred. (3) 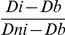 provides a means for comparing the amount of sequence divergence in the indel- and non-indel-containing haplotypes, where a value of 1 indicates no difference, and values greater than 1 indicate more divergence in the indel haplotype (see Materials and Methods).(0.06 MB DOC)

Table S4 The background nucleotide divergence (Db) for pairwise genome comparisons. The indel-associated increase in D extends only as far as window 2 (Figure 1A). Windows 3 through 10 were observed to be outside the range of influence of indel/region-associated increase in nucleotide substitution rate. Thus, the average D for these windows was used as an approximation of the background nucleotide divergence. For each *E. coli* two-strain comparison, Db was calculated by averaging the value of D over windows 3 to 10. These groups (each corresponding to a specific two-genome alignment) were compared using Tukey's HSD, which designates levels to each group. Two groups that do not share a letter are significantly different (Tukey's HSD, *p* < 0.05) in Db.
(0.04 MB DOC)

Table S5 Repeat sequence abundance can be used to identify regions with elevated nucleotide diversity. Shown are the results for the comparison of *E. coli* strains SE11 and REL606; these results are plotted in Figure 6. Highlighted in bold are those *p* values indicating a significant difference between the level of D for categories with a given number of repeats per window when compared to windows with zero repeats, as determined by Wilcoxon Sum Rank test (*p* < 0.05).
(0.03 MB DOC)

Table S6 Bacterial strains and analysed results used for Figure S4. The original data were from [6].
(0.06 MB DOC)

## Abbreviations

D: nucleotide diversity
Db: background divergence
Indel: insertion/deletion mutation
