# Supplementary material for: Clusters of Nucleotide Substitutions and Insertion/Deletion Mutations Are Associated with Repeat Sequences
Source: PLoS Biol. 2011 Jun 14;9(6):e1000622. doi: 10.1371/journal.pbio.1000622 (PMC3114760; doi:10.1371/journal.pbio.1000622)
Supplement: Table S1 — Bacterial and yeast strains used in this study. (A) Genomes of Escherichia coli strains used in this study. (B) Yeast strains used in this study. The accession number is given according to the internal collection at the University of Nottingham. Strains are grouped into geographic locations from which they were isolated (for a detailed phylogeny of S. paradoxus strains used in this study, see Liti et al. 2009 [14]). (0.05 MB DOC) [file pbio.1000622.s007.doc]

**Table S1a**

| **Ref seq ID** | **Strains** |
| --- | --- |
| NC_000913 | Escherichia coli str. K-12 substr. MG1655 |
| AC_000091 | Escherichia coli W3110 |
| NC_002655 | Escherichia coli O157:H7 EDL933 |
| NC_002695 | Escherichia coli O157:H7 str. Sakai |
| NC_007613 | Shigella boydii Sb227 |
| NC_007384 | Shigella sonnei Ss046 |
| NC_004337 | Shigella flexneri 2a str. 301 |
| NC_004741 | Shigella flexneri 2a str. 2457T |
| NC_008563 | Escherichia coli APEC O1 |
| NC_007946 | Escherichia coli UTI89 |
| NC_004431 | Escherichia coli CFT073 |
| NC_008253.1 | Escherichia coli str. 536 |
| NC_011740 | Escherichia fergusonii ATCC 35469 |
| NC_011741 | Escherichia coli IAI1 |
| NC_011742 | Escherichia coli S88 |
| NC_011745 | Escherichia coli ED1a |
| NC_011748 | Escherichia coli 55989 |
| NC_011750 | Escherichia coli IAI39 |
| NC_011751 | Escherichia coli UMN026 |
| NC_007606.1 | Shigella dysenteriae 1 197 |
| NC 012967 | Escherichia coli B. str. REL606 |
| NC 011415.1 | Escherichia coli SE11 |
| AP009378 | Escherichia coli SE15 |

**Table S1b**

| **Accession No.** | **Strains** | **Geographic Location** |
| --- | --- | --- |
| 186/A | Saccharomyces paradoxus A4 | American |
| 273/A | Saccharomyces paradoxus UWOPS91-917.7 | Hawaiian |
| 20/A | Saccharomyces paradoxus UFRJ50791 | American |
| 32/A | Saccharomyces paradoxus DBVPG6304 | American |
| 137/A | Saccharomyces paradoxus IFO1804 | Far Eastern |
| 77/A | Saccharomyces paradoxus N-44 | European |
| 76/A | Saccharomyces paradoxus N-43 | European |
| 301/A | Saccharomyces paradoxus Z1 | European |
| 26/A | Saccharomyces paradoxus N-17 | European |
| 169/A | Saccharomyces paradoxus Q62.5 | European |
| 96 | Saccharomyces cerevisiae S288c | American |
