# Supplementary material for: Clusters of Nucleotide Substitutions and Insertion/Deletion Mutations Are Associated with Repeat Sequences
Source: PLoS Biol. 2011 Jun 14;9(6):e1000622. doi: 10.1371/journal.pbio.1000622 (PMC3114760; doi:10.1371/journal.pbio.1000622)
Supplement: Table S2 — The accumulated amount of nucleotide substitutions in indel haplotypes is rarely significantly higher than the amount in non-indel haplotypes in the sequence window closest to the indel (window 1). The values of D for the indel- and non-indel-containing haplotypes for window 1 were compared using the two-sample Kolmogorov-Smirnov test (n is the number of indel/non-indel pairs used in the analysis). Indel and non-indel haplotypes have elevated nucleotide divergence in window 1 as compared to the background level of divergence (Db). The values of D for window 3 were chosen to represent Db; this level was compared with the level in window 1 to determine if there was a significant increase in nucleotide substitutions for both the indel and non-indel haplotypes by performing two-sample Kolmogorov-Smirnov tests. Significant values for p (p < 0.05) are indicated in bold. n is the number of indel/non-indel pairs used in the analysis. (0.04 MB DOC) [file pbio.1000622.s008.doc]

**Table S2**

|  | |  | |  | **Di/Dni** | | | | **Di / Db** | | | | **Dni/ Db** | |
| --- | --- | --- | --- | --- | --- | --- | --- | --- | --- | --- | --- | --- | --- | --- |
| Aligned | Divergence | Outgroup | | n | ratio | *p* | | | ratio | *p* | | | ratio | *p* |
| CFT073/ED1a | 0.95% | IAI39 | 178 | | 1.24 | | 0.941 | | 1.73 | | **0.002** | | 1.46 | **0.012** |
| CFT073/ED1a |  | UMN026 | | 309 | 1.10 | | | 0.947 | 1.53 | | | **5.28E-06** | 1.28 | **3.10E-04** |
| EDL933/Sakai | 0.14% | Sd197 | | 78 | 1.32 | | | 0.999 | 1.13 | | | 0.403 | 1.16 | 0.151 |
| EDL933/Sakai |  | Sf2457T | | 109 | 1.17 | | | 0.999 | 1.61 | | | **0.007** | 1.37 | **0.057** |
| K12/O157 | 1.75% | IAI39 | | 263 | 1.02 | | | 0.432 | 1.60 | | | **1.44E-04** | 1.44 | **1.43E-06** |
| K12/O157 |  | UMN026 | | 454 | 1.04 | | | 0.24 | 1.85 | | | **1.10E-09** | 1.71 | **5.14E-07** |
