# Supplementary material for: Clusters of Nucleotide Substitutions and Insertion/Deletion Mutations Are Associated with Repeat Sequences
Source: PLoS Biol. 2011 Jun 14;9(6):e1000622. doi: 10.1371/journal.pbio.1000622 (PMC3114760; doi:10.1371/journal.pbio.1000622)
Supplement: Table S3 — Divergence values for indel and non-indel haplotypes. (1) D is the average divergence between the entire genomes of strains being compared. Di and Dni denote the divergence in sequence window 1 of the indel-containing and non-indel-containing haplotype, respectively. Db is the background level of diversity as measured by sequence windows 3 to 10. (2) Outgroups were used to determine in which of the aligned genomes the indel had occurred. (3) provides a means for comparing the amount of sequence divergence in the indel- and non-indel-containing haplotypes, where a value of 1 indicates no difference, and values greater than 1 indicate more divergence in the indel haplotype (see Materials and Methods). (0.06 MB DOC) [file pbio.1000622.s009.doc]

**Table S3**

| **Species** |  |  |  |  |  |  |  |
| --- | --- | --- | --- | --- | --- | --- | --- |
| E. coli/Shigella | comparison |  | outgroup |  |  |  |  |
|  | IAI1-55989 | 0.0066 | Sb227 | 0.0311 | 0.0226 | 0.0097 | 1.66 |
|  | IAI1-Sf8401 | 0.0132 | Sd197 | 0.0286 | 0.0257 | 0.0179 | 1.37 |
|  | IAI1-Sakai | 0.0173 | UMN026 | 0.0426 | 0.034 | 0.0218 | 1.71 |
|  | K12-UMN026 | 0.0213 | APEC01 | 0.0403 | 0.0379 | 0.018 | 1.41 |
|  | K12-IAI1 | 0.0124 | O157 Sakai | 0.039 | 0.025 | 0.013 | 2.15 |
|  | K12-Sakai | 0.0164 | UMN026 | 0.0471 | 0.0399 | 0.0175 | 1.32 |
|  | UTI89-S88 | 0.0018 | IAI39 | 0.0396 | 0.0297 | 0.022 | 2.3 |
|  | 536-UTI89 | 0.0089 | IAI39 | 0.0351 | 0.0282 | 0.0206 | 1.91 |
|  | UTI89-IAI39 | 0.0227 | UMN026 | 0.040 | 0.0379 | 0.0214 | 1.12 |
|  | Sakai-Sd197 | 0.0138 | K12 | 0.0305 | 0.025 | 0.0155 | 1.58 |
| S. paradoxus |  |  |  |  |  |  |  |
|  | A4-UFRJ | 0.0029 | UWO | 0.0332 | 0.032 | 0.0197 | 1.09 |
|  | DBVP-UFRJ | 0.0029 | UWO | 0.0306 | 0.0292 | 0.02 | 1.15 |
|  | Z1-N44 | 0.0123 | UWO | 0.0578 | 0.0599 | 0.0342 | 0.92 |
|  | Z1-N44 | 0.0123 | A4 | 0.0637 | 0.065 | 0.0353 | 0.96 |
|  | N17-IFO1804 | 0.0123 | A4 | 0.0627 | 0.0639 | 0.0353 | 0.96 |
|  | Z1-A4 | 0.10 | S288C | 0.159 | 0.159 | 0.109 | 1.0 |
|  | N43-Q625 | 0.10 | S288C | 0.142 | 0.143 | 0.103 | 0.97 |
| Drosophila |  |  |  |  |  |  |  |
|  | D.sim-D.sec | 0.0311 | D.melano. | 0.0625 | 0.0557 | 0.0397 | 1.43 |
|  | D.mel-D.mel  least diverged | 0.045 | D. sechelia | 0.1567 | 0.1475 | 0.045 | 1.08 |
|  | D.mel-D.mel  most diverged | 0.0542 | D. sechelia | 0.1266 | 0.1267 | 0.078 | 0.99 |
|  | Dsim-D.mel | 0.078 | D. yakuba | 0.112 | 0.111 | 0.077 | 1.03 |
|  | D.sim-D.yak | 0.22 | D. ananassae | 0.291 | 0.291 | 0.23 | 1.00 |
