# Supplementary material for: Clusters of Nucleotide Substitutions and Insertion/Deletion Mutations Are Associated with Repeat Sequences
Source: PLoS Biol. 2011 Jun 14;9(6):e1000622. doi: 10.1371/journal.pbio.1000622 (PMC3114760; doi:10.1371/journal.pbio.1000622)
Supplement: Table S4 — The background nucleotide divergence (Db) for pairwise genome comparisons. The indel-associated increase in D extends only as far as window 2 (Figure 1A). Windows 3 through 10 were observed to be outside the range of influence of indel/region-associated increase in nucleotide substitution rate. Thus, the average D for these windows was used as an approximation of the background nucleotide divergence. For each E. coli two-strain comparison, Db was calculated by averaging the value of D over windows 3 to 10. These groups (each corresponding to a specific two-genome alignment) were compared using Tukey's HSD, which designates levels to each group. Two groups that do not share a letter are significantly different (Tukey's HSD, p < 0.05) in Db. (0.04 MB DOC) [file pbio.1000622.s010.doc]

**Table S4**

| **Group** | **Level** | **Db** |
| --- | --- | --- |
| K12 vs APEC01 | A | 0.0258 |
| K12 vs UTI89 | AB | 0.0252 |
| K12 vs CFT073 | AB | 0.0250 |
| K12 vs ED1a | B | 0.0246 |
| K12 vs Sakai | C | 0.0175 |
| K12 vs EDL933 | C | 0.0170 |
| K12 vs Sf2457T | D | 0.0144 |
| K12 vs Ss046 | DE | 0.0143 |
| K12 vs Sb227 | DE | 0.0138 |
| K12 vs Sf301 | EF | 0.01338 |
| K12 vs IAI1 | FG | 0.01334 |
| K12 vs 55989 | G | 0.0129 |
| Sb227 vs Ss046 | H | 0.0104 |
| IAI1 vs 55989 | I | 0.0097 |
| CFT073 vs ED1a | I | 0.0095 |
| Sf301 vs Sf2457T | J | 0.0017 |
| UTI89 vs APEC01 | J | 0.0015 |
| EDL933 vs Sakai | J | 0.0014 |
