# Supplementary material for: Clusters of Nucleotide Substitutions and Insertion/Deletion Mutations Are Associated with Repeat Sequences
Source: PLoS Biol. 2011 Jun 14;9(6):e1000622. doi: 10.1371/journal.pbio.1000622 (PMC3114760; doi:10.1371/journal.pbio.1000622)
Supplement: Table S5 — Repeat sequence abundance can be used to identify regions with elevated nucleotide diversity. Shown are the results for the comparison of E. coli strains SE11 and REL606; these results are plotted in Figure 6. Highlighted in bold are those p values indicating a significant difference between the level of D for categories with a given number of repeats per window when compared to windows with zero repeats, as determined by Wilcoxon Sum Rank test (p < 0.05). (0.03 MB DOC) [file pbio.1000622.s011.doc]

Table S5

| **repeats per window** | **D** | **% transversions of total substitutions** | | **D(repeat)**  **D(non-repeat)** | |
| --- | --- | --- | --- | --- | --- |
| 0 | 0.012 | 13.1% | *p* | 1 | *p* |
| 1 | 0.012 | 13.9% | 0.973 | 1.01 | 0.681 |
| 2 | 0.013 | 15.0% | 0.531 | 1.06 | **8.15E-09** |
| 3 | 0.014 | 16.3% | **8.15E-09** | 1.12 | **6.69E-10** |
| 4 | 0.014 | 15.0% | **0.045** | 1.13 | **5.65E-09** |
| 5 | 0.019 | 20.4% | **1.63E-08** | 1.48 | **0.006** |
| 6 | 0.022 | 24.8% | **0.025** | 1.68 | 0.19 |
