# Supplementary material for: Clusters of Nucleotide Substitutions and Insertion/Deletion Mutations Are Associated with Repeat Sequences
Source: PLoS Biol. 2011 Jun 14;9(6):e1000622. doi: 10.1371/journal.pbio.1000622 (PMC3114760; doi:10.1371/journal.pbio.1000622)
Supplement: Table S6 — Bacterial strains and analysed results used for Figure S4. The original data were from [6]. (0.06 MB DOC) [file pbio.1000622.s012.doc]

**Table S6**

| **species** | **indel (%)** | **non-indel (%)** | **Di/Dni** | **divergence (%)** |
| --- | --- | --- | --- | --- |
| *X. campestris* | 2.21 | 1.73 | 1.277457 | 1.39 |
| *L. lactis* | 2.35 | 0.79 | 2.974684 | 1.87 |
| *P. aeruginosa* | 1.19 | 0.74 | 1.608108 | 1.08 |
| *S. enterica* | 1.6 | 0.83 | 1.927711 | 1.38 |
| *F. tularensis* | 0.43 | 0.35 | 1.228571 | 0.56 |
| *S. pneumoniae* | 2.75 | 0.6 | 4.583333 | 1.19 |
| *B. cereus* | 2.87 | 2.57 | 1.116732 | 7.14 |
| *P. putida* | 4.25 | 3.73 | 1.13941 | 7.06 |
| *E. coli* | 3.61 | 1.34 | 2.69403 | 1.56 |
| *C. perfringens* | 1.67 | 0.74 | 2.256757 | 1.35 |
| *C. jejuni* | 1.97 | 0.98 | 2.010204 | 1.89 |
| *s. aureus* | 2.93 | 1.38 | 2.123188 | 1.3 |
| *X. fastidiosa* | 2.29 | 1.38 | 1.65942 | 2.07 |
| *Y. pseudotuberculosis* | 1.99 | 0.39 | 5.102564 | 0.55 |
| *C. botulinum* | 3.32 | 1.46 | 2.273973 | 2.21 |
| *P. marinus* | 5.29 | 4.51 | 1.172949 | 9.56 |
| *H. pylori* | 4.3 | 2.35 | 1.829787 | 5.02 |
| *L. pneumophila* | 4.8 | 1.77 | 2.711864 | 2.71 |
| *R. palustris* | 6.49 | 5.82 | 1.11512 | 12.8 |
| *P. syringae* | 6.49 | 5.5 | 1.18 | 11.2 |
| *S. pyogenes* | 2.55 | 0.65 | 3.923077 | 1.11 |
| *A. pleuropneumoniae* | 4.52 | 0.39 | 11.58974 | 1.57 |
| *S. baltica* | 4.73 | 1.64 | 2.884146 | 2.86 |
| *H. influenzae* | 1.89 | 1.113 | 1.698113 | 2.44 |
| *N. meningitidis* | 5.53 | 0.99 | 5.585859 | 2.67 |
| *A. baumannii* | 2.6 | 0.87 | 2.988506 | 1.91 |
